# Structures and Functions of the 3′ Untranslated Regions of Positive-Sense Single-Stranded RNA Viruses Infecting Humans and Animals

**DOI:** 10.3389/fcimb.2020.00453

**Published:** 2020-08-27

**Authors:** Yuanzhi Liu, Yu Zhang, Mingshu Wang, Anchun Cheng, Qiao Yang, Ying Wu, Renyong Jia, Mafeng Liu, Dekang Zhu, Shun Chen, Shaqiu Zhang, XinXin Zhao, Juan Huang, Sai Mao, Xumin Ou, Qun Gao, Yin Wang, Zhiwen Xu, Zhengli Chen, Ling Zhu, Qihui Luo, Yunya Liu, Yanling Yu, Ling Zhang, Bin Tian, Leichang Pan, Xiaoyue Chen

**Affiliations:** ^1^Institute of Preventive Veterinary Medicine, Sichuan Agricultural University, Chengdu, China; ^2^Key Laboratory of Animal Disease and Human Health of Sichuan Province, Sichuan Agricultural University, Chengdu, China; ^3^Research Center of Avian Disease, College of Veterinary Medicine, Sichuan Agricultural University, Chengdu, China

**Keywords:** 3′ UTR, structures, functions, ssRNA(+), viruses

## Abstract

The 3′ untranslated region (3′ UTR) of positive-sense single-stranded RNA [ssRNA(+)] viruses is highly structured. Multiple elements in the region interact with other nucleotides and proteins of viral and cellular origin to regulate various aspects of the virus life cycle such as replication, translation, and the host-cell response. This review attempts to summarize the primary and higher order structures identified in the 3′UTR of ssRNA(+) viruses and their functional roles.

## Introduction

**Figure 1:**
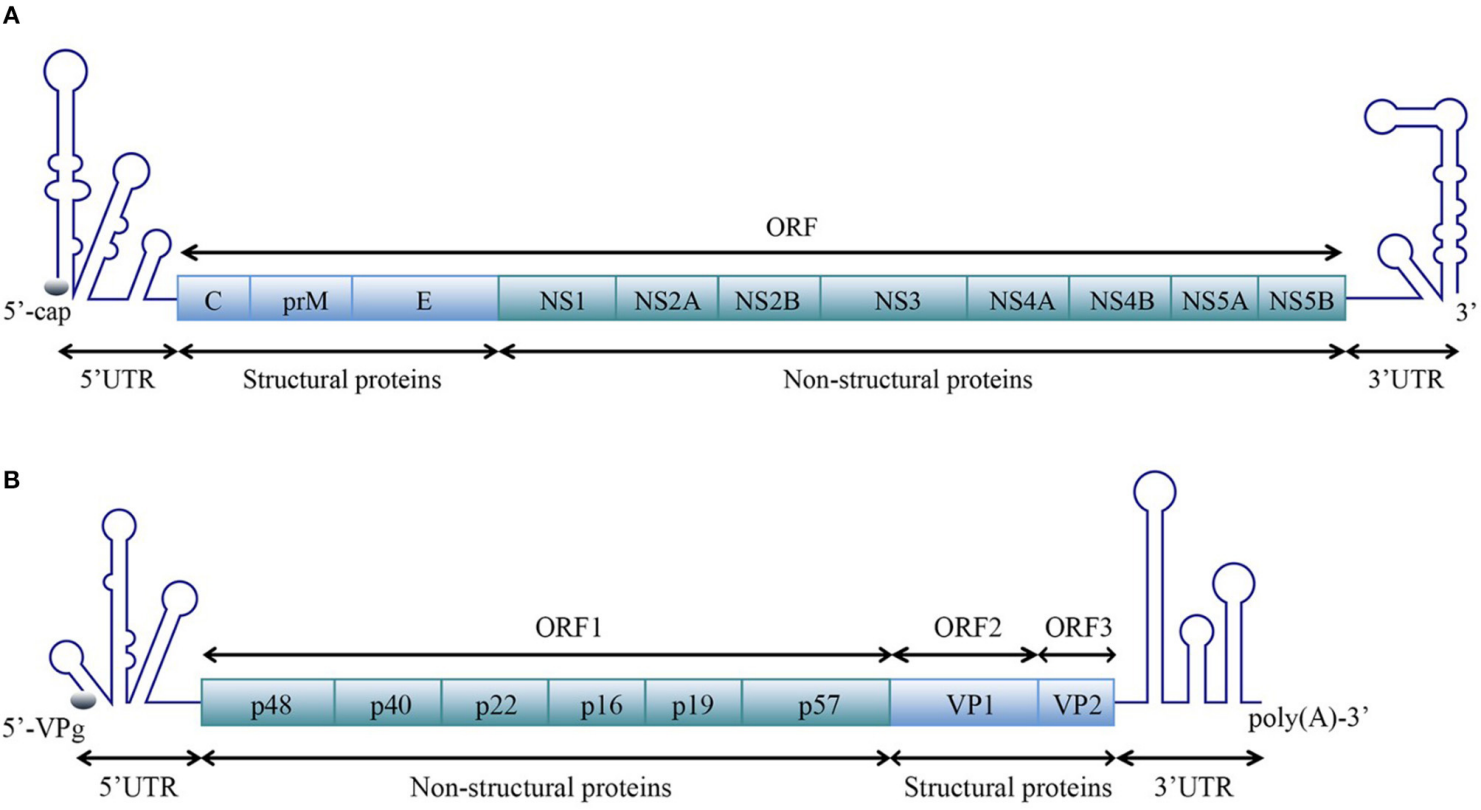
Schematic representation of the organization of the ssRNA(+) virus genome. A typical ssRNA(+) virus genomic RNA is comprised of a 5′ UTR, ORF(s), and a 3′ UTR. **(A)** Viral genome with one open reading frame (ORF), using the example of WNV in the *Flaviviridae* family [reviewed in Suthar et al. (2013)]. **(B)** Viral genome with three ORFs, using the example of norovirus (NV) in the *Caliciviridae* family [reviewed in Thorne and Goodfellow (2014)]. The diagrams do not represent the true genomic length and structure.

Positive-sense single-stranded RNA [ssRNA(+)] viruses consist of 56 families according to the current ICTV (International Committee on Taxonomy of Viruses) Report on Virus Taxonomy (International Committee on Taxonomy of Viruses, 2019). The genomic RNAs of all ssRNA(+) viruses function as mRNAs and are directly translated to produce one or more polyproteins. A typical ssRNA(+) virus genomic RNA is comprised of a 5′ untranslated region (5′ UTR), one or more open reading frames (ORFs) and a 3′ UTR. For most ssRNA(+) viruses, the 5′-terminus of the genomic RNA is covalently linked to a small VPg protein or a protein equivalent to VPg or a cap structure, and the 3′-terminus is polyadenylated or the 3′ UTR contains poly(A/U) regions, similar to the polyadenylation signal (Figure 1).

Once a ssRNA(+) virus invaded a host cell, its RNA will first attach to the host ribosome and be translated to produce one or more polyproteins, which will be rapidly cleaved by viral and/or cellular enzymes to generate several structural and non-structural proteins that participate in assembling complete virus particles. RNA-dependent RNA polymerase (RdRP), a virus encoded non-structural protein, synthesizes negative-strand RNAs using viral positive-strand RNAs as templates. Subsequently, more positive-stranded RNAs will be synthesized by RdRP using negative-strand RNAs as templates. However, for viruses of the order *Nidovirales*, both the synthesis of positive-stranded genomic RNAs and the transcription of subgenomic mRNA (sg mRNA) are required for viral replication. The sg mRNA contains same 5′ and 3′-termini and will be translated to produce different structural proteins (Pasternak et al., 2006; Cao et al., 2012, 2013, 2016; Sun et al., 2016, 2017; Ou et al., 2017, 2018; Lai et al., 2019; Wen et al., 2019).

Both the synthesis of negative-strand RNA and the translation of viral polyproteins use positive-strand RNA as a template. How to reasonably convert these two processes is a problem encountered by all ssRNA(+) viruses. Taking poliovirus as an example, poly(rC)-binding protein 2 (PCBP2) is required for both IRES-dependent translation and RNA replication. When performing IRES-dependent translation, PCBP2 interacts with the IRES element and recruits ribosomes to viral RNA, then the ribosomes that move from 5′ to 3′ block RdRP 3D that synthesizes negative stranded RNA from 3′ to 5′. As the viral protease 3C/3CD accumulates, PCBP2 is cleaved by 3C/3CD, which blocks the recruitment of ribosomes to the IRES element. At this time, RdRP 3D can successfully pass through the RNA and synthesize negative-strand RNA from 3′ to 5′ (Perera et al., 2007).

Similar to the 3′ UTR of the mature eukaryotic mRNA, the 3′ UTR of the ssRNA(+) virus genomic RNA possess multiple functional primary and higher order structures. These structures have been predicted through bioinformatics analyses and validated experimentally. Although the nucleotide sequences of the 3′ UTR from viruses of same species differ between strains, their higher order structures are generally conserved. Nucleotides and proteins from the virus and host directly or indirectly interact with structural elements in 3′ UTRs, participating in regulating the cyclization, replication, and translation of viral genomic RNA (Tables 1, 2); studies of the structure and function of the 3′ UTRs of ssRNA(+) viruses are important for clarifying the detailed mechanism of the viral life cycle and anti-viral research.

**Table 1:** Cellular proteins that interact with the 3′ UTRs of ssRNA(+) viruses.

**Host protein**	**Virus**	**Binding site**	**Function in viral replication**	**References**
PABP	DENV-2	(+)3′ DB	Required for viral RNA circularization, replication, and translation	Polacek et al. (2009b)
	PV, NV	(+)3′ poly(A) tail		Herold and Andino (2001); Gutiérrez-Escolano et al. (2003)
	TGEV	(+)3′ UTR		Galán et al. (2009)
PCBP1, PCBP2	MNV	(+)3′ p(Y)	Involved in viral replication and virulence	Bailey et al. (2010)
	HCV	(+)3′ UTR		Tingting et al. (2006)
PTB	MHV	nt 53 to 149 of (-)c3′ UTR	Induces viral RNA conformation changes and enhances virus replication and translatin	Huang and Lai (1999)
	HCV	(+)3′ X		Ito and Lai (1999); Luo (1999); Chang and Luo (2006)
	DENV-4, NV, SHFV	(+)3′ SL		Herold and Andino (2001); De Nova-Ocampo et al. (2002); Gutiérrez-Escolano et al. (2003); Maines et al. (2005)
	JEV	(-)3′ SL		Kim and Jeong (2006)
	MNV	(+)3′ p(Y)		Bailey et al. (2010)
	HAstV-8, CVB3	(+)3′ UTR		Verma et al. (2010); Espinosa-Hernandez et al. (2014)
FBA-A	SHFV	(+)3′ SL	Plays roles in viral replication and virulence	Maines et al. (2005)
DDX1	HCV	(+)3′ UTR	Unknown	Tingting et al. (2006)
DDX5	JEV	(+)3′ UTR	Enhances virus replication	Li et al. (2013)
DDX6	DENV-2	(+)3′ DB	Required for infectious virus production	Ward et al. (2011)
eEF1A	WNV, DENV-4	(+)3′ SL	Enhances virus replication	Blackwell and Brinton (1997); De Nova-Ocampo et al. (2002); Davis et al. (2007)
La	JEV, NV	(+)3′ SL	Required for viral RNA replication and translation	Herold and Andino (2001); Gutiérrez-Escolano et al. (2003); Vashist et al. (2009, 2011)
	HCV	(+)3′ UTR	Protects viral RNA from degradation and enhances viral RNA replication	Spangberg et al. (2001); Kumar et al. (2013)
	DENV-4	(+)3′ SL, (-)c3′ UTR	Required for viral RNA replication	De Nova-Ocampo et al. (2002); Yocupicio-Monroy et al. (2003, 2007); Garcia-Montalvo et al. (2004)
LSm1	DENV-2	(+)3′ UTR	Enhances viral RNA translation and replication	Dong et al. (2015)
	HCV	(+)5′ UTR, (+)3′ UTR		Scheller et al. (2009)
L22	HCV	(+)3′ X	Enhances viral RNA translation	Wood et al. (2001)
NF90, NF110, NF45, RHA	DENV-2	(+)3′ SL	Required for viral RNA cyclization, replication, and translation	Gomila et al. (2011)
	BVDV	(+)3′ V		Isken et al. (2003, 2004)
	HCV	(+)3′ UTR		Isken et al. (2007)
p100(NFKB2)	DENV-2	(+)3′ SL	Required for viral RNA replication	Lei et al. (2011)
TIAR, TIA-1	WNV, DENV-2	(-)c3′ SL	Required for viral RNA replication	Li et al. (2002); Emara and Brinton (2007); Emara et al. (2008)
G3BP1, TIA1, HUR	EV-D68	(+)3′ UTR	Inhibits viral replication by chelating the viral RNA	Cheng et al. (2020)
HuR	HCV	(+)3′ UTR, (-)3′ UTR	Protects the viral RNA from degradation	Spångberg et al. (2000)
	CSFV	(+)3′ ARE	Unknown	Nadar et al. (2011)
Nucleolin	FCV, NV	(+)3′ UTR	Required for virus replication	Cancio-Lonches et al. (2011)
	PV	(+)3′ UTR	Required for infectious virus production	Waggoner and Sarnow (1998)
AUF1	CVB3	(+)3′ UTR	Enhances viral genome stability	Wong et al. (2013)
40S ribosomal subunit	HCV	(+)3′ UTR	Regulates viral RNA translation	Bai et al. (2013)
hnRNPA1	MHV	(+)3′ UTR	Mediates potential 5′-3′-end cross-talk	Huang and Lai (2001)
hnRNPA2	JEV	(-)c3′ UTR	Required for viral RNA replication	Katoh et al. (2011)
hnRNPC	HCV	(+)3′ UTR, (-)3′ UTR	Protects the viral RNA from degradation	Spångberg et al. (2000)
hnRNPE2	HCV	(+)3′ UTR	Unknown	Tingting et al. (2006)
hnRNPQ	TGEV	(+)3′ UTR	Enhances viral RNA replication	Galán et al. (2009)
EPRS	TGEV	(+)3′ UTR	Enhances viral RNA replication	Galán et al. (2009)
Mov34	JEV	(+)3′ SL	May play roles in viral RNA replication	Ta and Vrati (2000)
m-aconitase	MHV	(+)3′ UTR	Unknown	Nanda and Leibowitz (2001); Nanda et al. (2004)
FBP1	JEV	(+)3′ UTR	Inhibits the translation of viral RNAs	Chien et al. (2011)
G3BP1, G3BP2, CAPRIN1	DENV-2	(+)3′ VR	Relevant to virus replication and pathogenesis	Bidet et al. (2014)
	HCV	(+)3′ UTR	Regulates virus replication	Tingting et al. (2006)
GAPDH	TGEV	(+)3′ UTR	Inhibits viral RNA replication	Galán et al. (2009)
YBX1	DENV-2	(+)3′ SL	Inhibits viral RNA translation	Paranjape and Harris (2007)
MCPIP1	DENV-2, JEV	(+)3′ UTR	Degrades viral RNAs	Lin et al. (2013)

*PABP, Poly(A)-binding protein; DENV-2, Dengue virus type 2; DB, Dumbbell; PV, Poliovirus; NV, Norovirus; TGEV, Transmissible gastroenteritis coronavirus; UTR, Untranslated region; PCBP, Poly(C)-binding protein; MNV, Murine norovirus; p(Y), Polypyrimidine tract; HCV, Hepatitis C virus; PTB, Polypyrimidine tract-binding protein; 3′ X, 3′ X-region; MHV, Mouse hepatitis virus; DENV-4, Dengue virus type 4; SHFV, Simian hemorrhagic fever virus; SL, Stem loop; JEV, Japanese encephalitis virus; HAstV-8, Human astrovirus type 8; CVB3, Coxsackievirus B3; BVDV, Bovine viral diarrhea virus; CSFV, Classical swine fever virus; FCV, Feline calicivirus; FBA-A, Fructose bisphosphate aldolase A; DDX, DEAD (D-E-A-D: Asp-Glu-Ala-Asp)-box; eEF1A, Eukaryotic elongation factor 1A; NF, Nuclear factor; RHA, RNA helicase A; NFKB, NF-kappa B; 3′ V, 3′ variable region; TIAR, T-cell intracellular antigen-related protein; TIA-1, T-cell intracellular antigen-1; HuR, Hu antigen R; AUF1, Adenosine-uridine-rich element RNA binding factor 1; hnRNP, Heterogeneous nuclear ribonucleoprotein; EPRS, Glutamyl-prolyl-tRNA synthetase; FBP1, FUSE (far upstream element) binding protein 1; GAPDH, Glyceraldehyde 3-phosphate dehydrogenase; YBX1, Y box-binding protein-1; MCPIP1, Monocyte chemoattractant protein 1-induced protein 1*.

**Table 2:** Viral proteins that interact with the 3′ UTRs of ssRNA(+) viruses.

**Virus protein**	**Virus**	**Binding site**	**Function in viral replication**	**References**
CP	HCV	(+)3′ SL	Unknown	Yu et al. (2009)
3D	EMCV	(+)3′ UTR	Initiates viral RNA synthesis	Cui et al. (1993)
NS5A, NS5B	CSFV	(+)3′ SL1, (+)3′ SL2, (-)3′ UTR	Regulates viral RNA replication	Sheng et al. (2007, 2012); Chen et al. (2012)
NS5A	HCV	polyU/UC	Inhibits viral RNA translation	Hoffman et al. (2015)
Nucleocapsid	IBV	(+)3′ UTR	Unknown	Collisson et al. (2001)
NS2A	KUNV	(+)3′ UTR	Unknown	Mackenzie et al. (1998)
	DENV-2	pk3, pk4, and 3′SL of (+)3′ UTR	The signal to recruit viral RNA to the virion assembly site	Xie et al. (2019)

*CP, Core protein; EMCV, Encephalomyocarditis virus; NS5A, Non-structural proteins 5A; NS5B, Non-structural proteins 5B; KUNV, Kunjin virus*.

## Primary Structures and Functions

Primary structures, or nucleotide sequences, are the basis for the formation of specific higher-order structures. For ssRNA(+) viruses, their genomic RNAs contain several functional sequences, which directly participate in viral genome cyclization, replication, and translation and have important functions in the viral life cycle.

### Cyclization Sequences and Genome Cyclization of Flaviviruses

**Figure 2:**
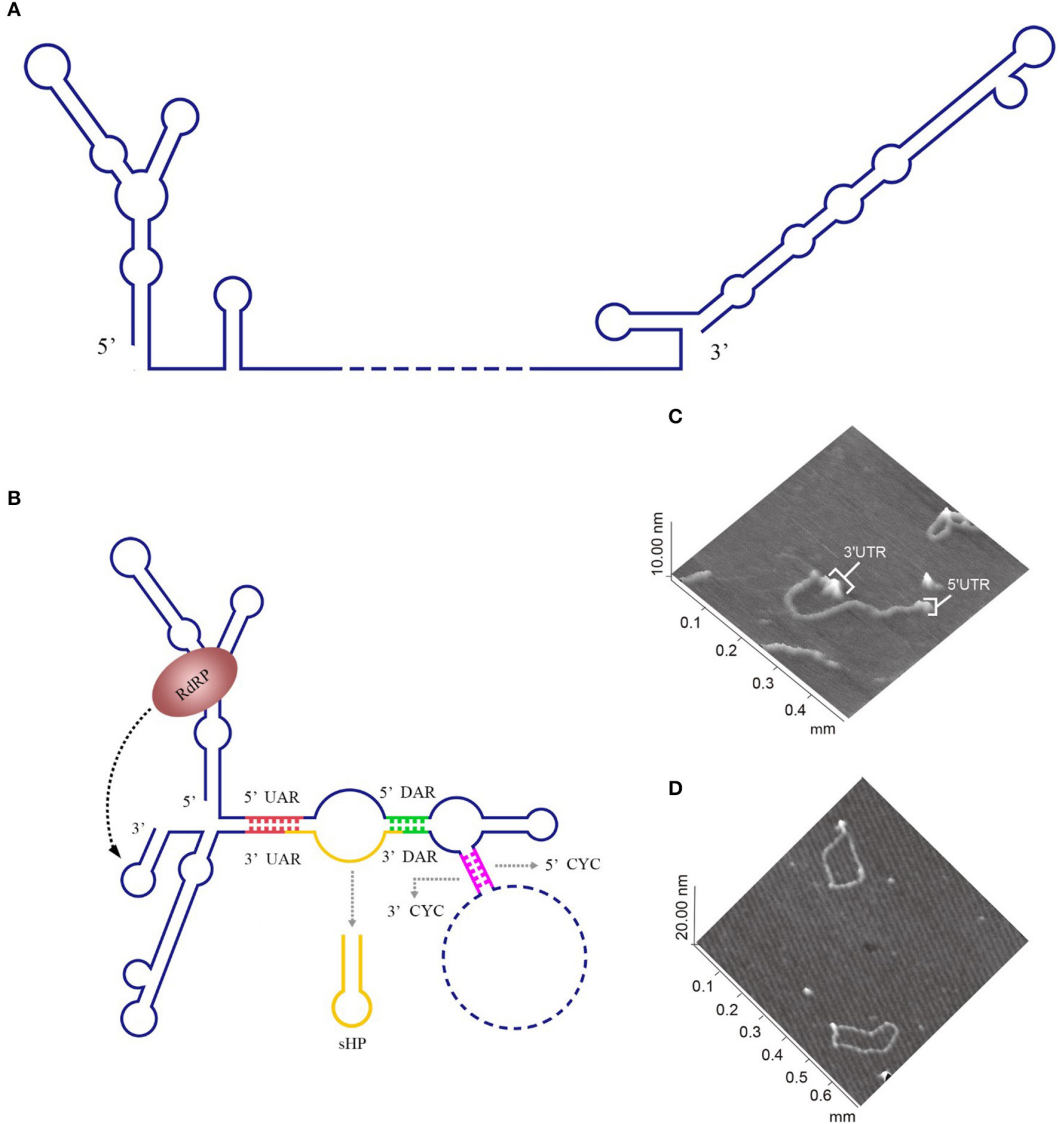
Circularization of the DENV genome. **(A)** The linear form of the DENV genomic RNA. When the interactions between the 3′ and 5′cyclization sequences are not considered, the DENV genome appears in a linear form (Villordo and Gamarnik, 2009). **(B)** The circularized form of the DENV genomic RNA. 5′ CYC/3′ CYC, 5′ UAR/3′ UAR, and 5′ DAR/3′ DAR bind complementarily and cyclize the DENV genome, reducing the distance between the 5′ and 3′ ends and exposing the 3′-terminal nucleotides, which are indispensable for RdRP to recognize the 3′-terminus and initiate negative-strand synthesis [reviewed in Nicholson and White (2014)]. **(C)** Visualization of the model RNA molecules using AFM. A single RNA molecule is shown in a linear conformation. The double-stranded RNA region is flanked by single-stranded regions corresponding to the 5′ UTR and 3′ UTR of dengue virus. **(D)** Image of individual RNA molecules in the circular conformation. Contacts between the 5′ and 3′ single-stranded regions of the molecules are observed. The images shown in **(C)** and **(D)** were excerpted from a previous study (Alvarez et al., 2005) after obtaining the authors' permission. Figures have been approved by the original author and obtained the licenses through Rights Licensing Expert (www.copyright.com), the license numbers were, respectively 4045761041172 (for **A,B**) 4040580733464 (for **C,D**).

For members of the *Flavivirus* genus in the *Flaviviridae* family, replication of genomic RNAs includes the cyclization process. A long-range RNA-RNA interaction occurs when a short sequence of the 3′ UTR complements with another short sequence of the 5′ UTR and the 5′ UTR' s contiguous upstream, which cyclizes the genomic RNA [reviewed in Nicholson and White (2014)]. Genome cyclization has been observed with atomic force microscopy (AFM) (Figures 2C,D).

**Figure 3:**
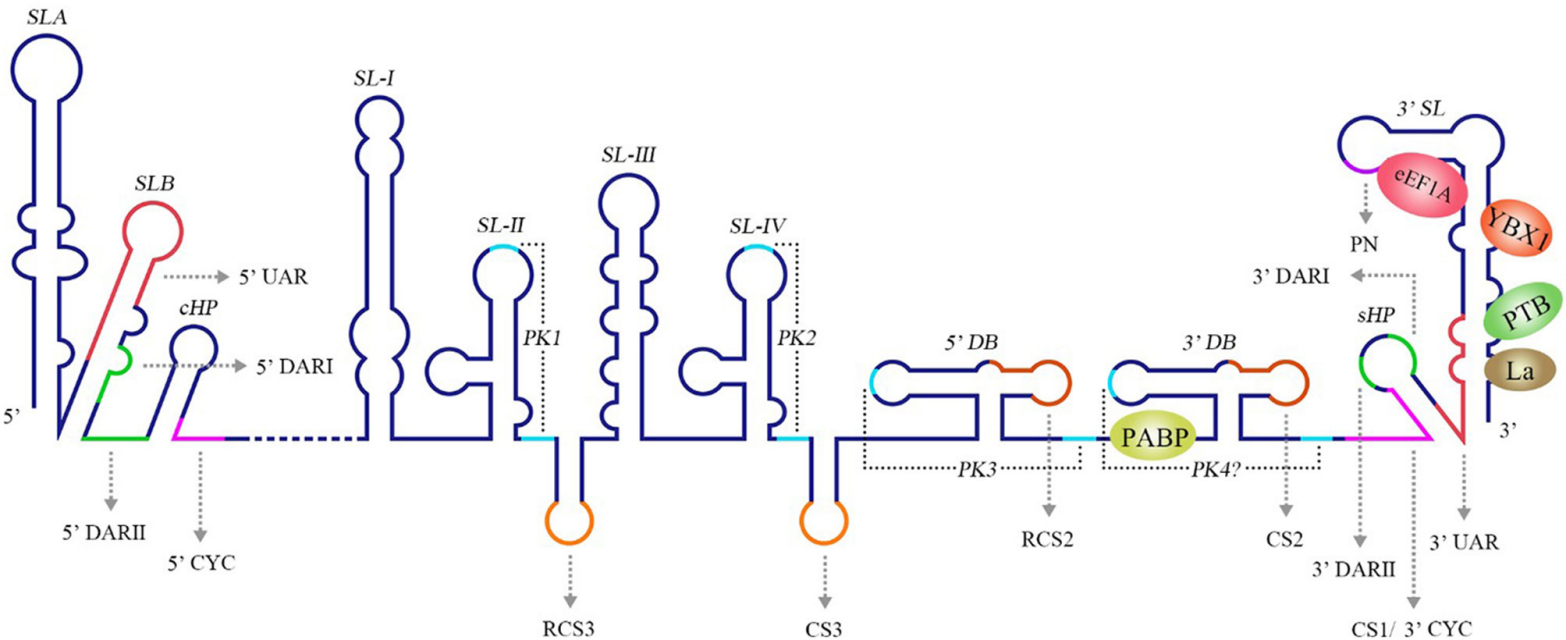
Structure of the WNV genomic RNA. The arrows represent WNV circularization sequences and other conserved sequences, italics indicate higher order structures of the WNV RNA, and proteins that bind to WNV 3′ UTR are shown in ovals with different colors [reviewed in Bidet and Garcia-Blanco (2014)]. SL, Stem-loop; UAR, Upstream AUG region; DAR, Downstream AUG region; CYC, Cyclization sequence; cHP, Capsid hairpin; sHP, Small hairpin structure; RCS, Repeat conserved sequence; PK, Pseudoknot; DB, Dumbbell; PN, 5′-CACAG-3′ pentanucleotide; eEF1A, Eukaryotic elongation factor 1A; YBX1, Y box-binding protein-1; PABP, Poly(A)-binding protein; PTB, Polypyrimidine tract-binding protein.

The 3′ UTR in the flavivirus genome possess a stem-loop termed the 3′ SL containing conserved sequence 1 (CS1) in which an 8 nt-long 3′ cyclization sequence (3′ CYC) is located (Hahn et al., 1987). The 3′ CYC complementarily binds to a 5′ CYC located in the capsid protein coding region (Figure 3). Research into dengue virus (DENV), West Nile virus (WNV) and tick-borne encephalitis virus (TBEV) has shown that the interaction between the 3′ CYC and 5′ CYC and several base sites among them are required for viral RNA replication, and the precise and specific sequences determine the function of the CYC, but not the secondary structure and complementary relationship between base sites (Kofler et al., 2006; Suzuki et al., 2008; Basu and Brinton, 2011; Manzano et al., 2011). However, the 5′ CYC-3′ CYC interaction of DENV does not participate in viral translation (Holden et al., 2006). In addition to the 5′ CYC/3′ CYC, the upstream AUG region (UAR) and downstream AUG region (DAR) also function as cyclization sequences involved in viral RNA cyclization. The 5′ UAR is located in immediately upstream of the start codon AUG, while the 3′ UAR is located at the bottom of the 3′ SL (Figure 3). The 5′ UAR-3′ UAR complementary relationship, which is stabilized by the 5′ CYC-3′ CYC interaction (Polacek et al., 2009a), is necessary for viral replication. Similarly, the 5′ UAR/3′ UAR interaction rarely affects viral translation (Zhang et al., 2008). The 5′ DAR is located immediately downstream of the start codon AUG, and the 3′ DAR is located at the bottom of the hairpin stem-loop termed HP-3′ SL (Figure 3). The interaction between the 5′ DAR and 3′ DAR is involved in viral RdRP reactivity and RNA replication efficiency (Friebe et al., 2011), but further studies are needed to determine whether they are also irrelevant to translation.

The 5′ CYC/3′ CYC, 5′ UAR/3′ UAR, and 5′ DAR/3′ DAR form several base pairs within the flavivirus genome that enable the linear genomic RNA to transform to a cyclized form. According to a phylogenetic analysis, the cyclization sequences are highly conserved among mosquito-borne flavivirus genomes (Zhang et al., 2008), indicating that the long-range RNA-RNA interactions between the 5′ UTR and 3′ UTR are very likely the common mechanism of flavivirus genome cyclization. In addition, DENV RdRP binds a stem-loop SLA in the 5′ UTR of the genome and facilitates viral replication by forming interactions between the cyclization sequences of 5′ UTR and 3′ UTR (Filomatori et al., 2006; Dong et al., 2007). Thus, the binding of RdRP to SLA, and the complementary pairing between cyclization sequences may have some connection in the replication of DENV RNA. A relatively complete and rational model was established to explain the balance between circular and linear forms of the DENV genome and the initiation of RNA synthesis by RdRP (Villordo and Gamarnik, 2009) (Figures 2A,B). In this model, 5′ CYC/3′ CYC, 5′ UAR/3′ UAR, and 5′ DAR/3′ DAR complement and cyclize the DENV genome, shortening the distance between 5′ and 3′ ends and exposing the 3′-terminal nucleotides. Once RdRP binds to SLA, its position will be near the 3′-terminus, which allows the RdRP to recognize the 3′-terminus and initiate negative-strand synthesis. Moreover, the WNV 3′ UAR could alternate between pairing with 5′ UAR or with the local 3′ end (Zhang et al., 2008) and RdRP binds to the 5′ DAR (Dong et al., 2008), which may be also involved in cyclizing the viral genome. The flaviviral genome cyclization mechanism and the initiation of the negative-strand synthesis have been extensively studied; however, the synthesis of positive-strand RNAs from negative-strand RNAs is still poorly understood and more information is needed to elucidate the complete mechanism of cyclization and synthesis of the flavivirus genome.

### Other Specific Sequences With Important Functions

A conserved 5′-CACAG-3′ pentanucleotide (PN) located in the 3′ SL of the flavivirus 3′ UTR (Figure 3) is required for viral replication, but not translation (Khromykh et al., 2003; Elghonemy et al., 2005; Tilgner et al., 2005). However, only the G in the 5′-CACAG-3′ sequence is required for yellow fever virus (YFV) replication (Silva et al., 2007). In addition to this PN, a conserved 5′-ACAGUGC-3′ sequence in the flavivirus 3′ SL may be involved in formation of the viral replication complex and plays a role in viral replication (Khromykh et al., 2003; Elghonemy et al., 2005). The specific 5′-CACAG-3′ and 5′-ACAGUGC-3′ nucleotides may interact with other sequences or proteins of the virus to regulate viral replication, but additional in-depth studies are required.

Research examining the Japanese encephalitis virus (JEV), Kunjin virus (KUNV), WNV, DENV, and YFV has revealed multiple conserved sequences in the flaviviral 3′ UTR, including conserved sequence 1 (CS1), CS2, CS3 and repeat conserved sequence 2 (RCS2), RCS3 (Figure 3); these sequences are required for efficient viral replication and translation (Hahn et al., 1987; Khromykh and Westaway, 1997; Wei et al., 2009).

## High-Order Structures and Functions

### Stem-Loop Structures

Stem-loop structures consist of stem regions and loop regions and are widely present in the secondary structures of single-stranded RNA. The stem is the double-stranded region formed by base pairings between the reverse complementary sequence, and the loop is the single-stranded region formed by unpaired bases. By interacting with viral and host proteins, stem-loop structures regulate viral replication and translation and are multifunctional and important for adjusting the life activities of the virus and host, as shown in Table 1. Here, the widely studied stem-loop structures and the corresponding functions are discussed with their interacting proteins.

**Figure 4:**
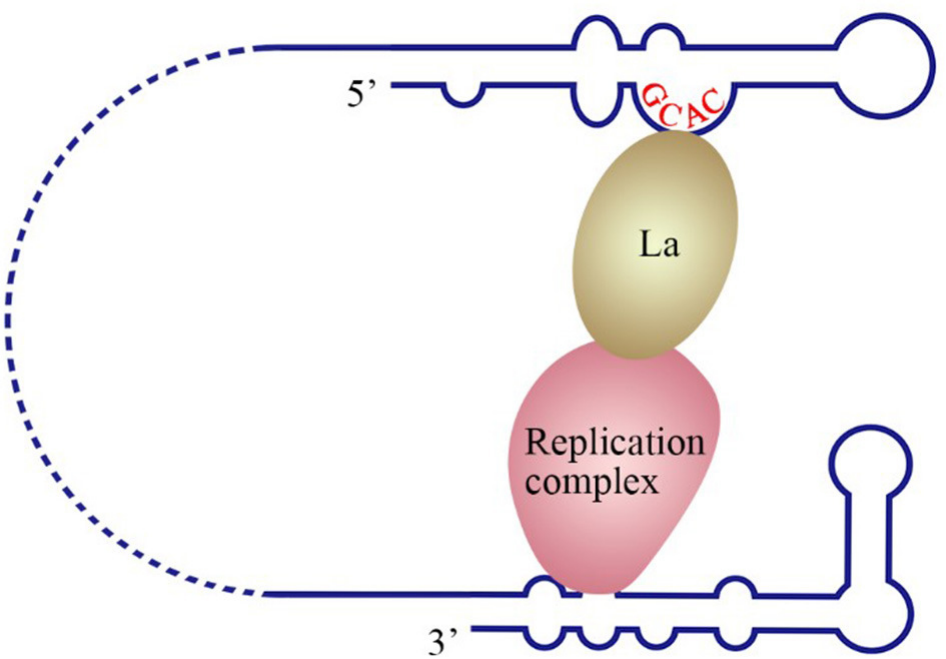
Model of the initiation of HCV replication. La interacts with the GCAC motif within the HCV IRES, and the replication complex interacts with the 3′ UTR. The interaction between La and the replication complex cyclizes the HCV genome and promotes 5′ to 3′ communication in favor of viral negative-strand synthesis (Kumar et al., 2013).

**Figure 5:**
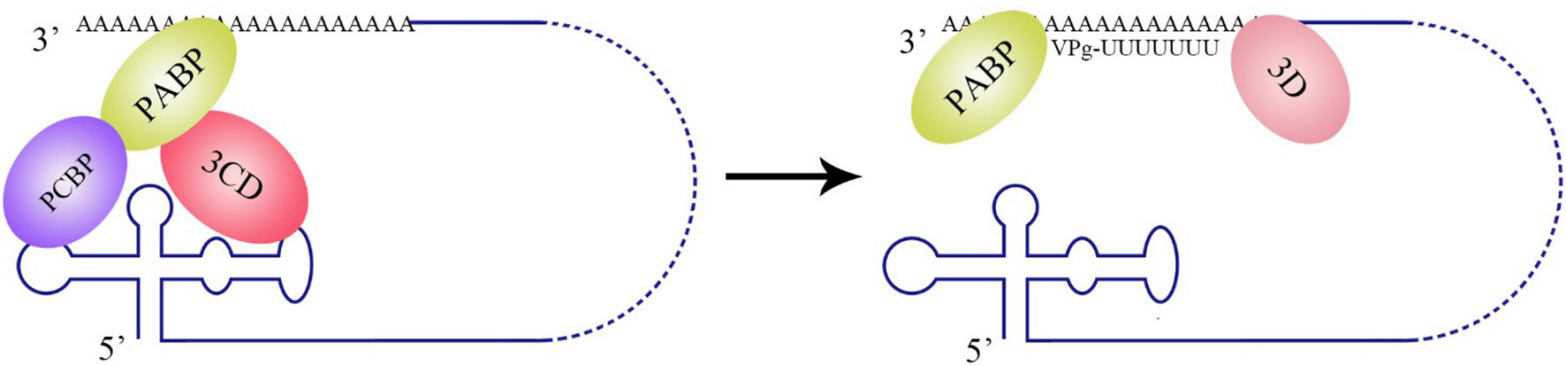
Circularization of the PV genome and initiation of negative-strand RNA synthesis. PV polypeptide and cellular factor poly(C)-binding protein (PCBP) bind to the 5′ UTR cloverleaf, and cellular factor poly(A)-binding protein (PABP) binds to the 3′ UTR. 3CD, PCBP, and PABP interact with each other and lead to the cyclization of the genomic RNA, which brings the viral RdRP (3D protein) in close proximity to the 3′ poly(A) tail and facilitates the initiation of negative-strand RNA synthesis (Herold and Andino, 2001).

The La protein binds to the 3′ UTRs of most members of the *Flaviviridae* family (Herold and Andino, 2001; Spangberg et al., 2001; De Nova-Ocampo et al., 2002; Garcia-Montalvo et al., 2004; Vashist et al., 2009). The binding site for La in the DENV-4 3′ UTR are located in the area between CS1 and 3′ SL (De Nova-Ocampo et al., 2002). Additionally, La binds to the DENV-encoded non-structural proteins NS3 and NS5 (Garcia-Montalvo et al., 2004), which possess protease and RdRP activity, respectively. Interestingly, the binding of the La protein from mosquito cells to 3′ UTRs of the positive- and negative-strand of DENV-4 inhibits the synthesis of the positive- and negative-strand of the genome (Yocupicio-Monroy et al., 2007). This may be related to the adaptability of the host. La proteins from human and mosquitoe may have different ability to regulate viral replication. La protein from mosquitoe may control the viral load in its body, making it better as a vector to infect human. Moreover, the binding of La to HCV 3′ UTR protects the 3′ UTR from degradation by the cytoplasmic RNase and mediates the circularization of HCV genome and viral replication, which are enhanced by the interaction between HuR and La (Spangberg et al., 2001; Shwetha et al., 2015). La also binds to a 5′-GCAC-3′ sequence in the HCV 5′ UTR, and mutation of 5′-GCAC-3′ influences the binding of La to viral RdRP NS5B and inhibits viral replication, but translation is not affected (Kumar et al., 2013). Thus, a model was proposed to explain the functions of La and the 5′-GCAC-3′ sequence in HCV replication (Kumar et al., 2013). NS5 and 3′ UTR form a virus replication complex, and La binding with NS5 and 5′-GCAC-3′ brings the 5′ UTR closer to the 3′ UTR (Figure 4). Interestingly, a cyclized conformation of the HCV genome is observed in this model, which resembles the genome cyclization strategy used by poliovirus (PV). The PV RdRP containing the precursor protein 3CD and the cellular factor PCBP bind to the 5′ UTR cloverleaf, and the cellular factor poly(A)-binding protein (PABP) binds to the 3′ UTR. Interactions between 3D, PCBP, and PABP hold the 5′ and the 3′ ends of the PV RNA in a non-covalent juxtaposition that leads to the circularization of the genomic RNA. These interactions bring the viral RdRP (3D protein) in close proximity to the 3′ poly(A) tail and enable the initiation of negative-strand RNA synthesis (Herold and Andino, 2001) (Figure 5). Furthermore, transcription factor NF90 also participates in cyclizing the HCV and bovine viral diarrhea virus (BVDV) genome (Isken et al., 2003). Thus, in addition to long-range RNA-RNA interactions, protein-protein and protein-RNA interactions are also relevant to viral genome cyclization.

The genomic 3′ UTR of ssRNA(+) virus usually contains one or more polyadenylated regions, which have been widely reported to interact with the cellular polypyrimidine tract-binding protein (PTB). PTB binds to 3′ SL of HCV (Ito and Lai, 1999; Luo, 1999; Chang and Luo, 2006), DENV4 (De Nova-Ocampo et al., 2002), simian hemorrhagic fever virus (SHFV) (Maines et al., 2005) and mouse hepatitis virus (MHV) (Huang and Lai, 1999), to the NV (Gutiérrez-Escolano et al., 2003) positive-strand RNA and to the 3′ SL of the JEV (Kim and Jeong, 2006) negative-strand RNA. Mutation of the PTB binding site in the CVB3 3′ UTR reduces viral translation, indicating that PTB is involved in CVB3 translation (Verma et al., 2010). According to a similar study, the binding of PTB to the HCV 3′ X region in the 3′ UTR may stimulate viral replication and translation (Brocard et al., 2007), which is associated with the competition between HuR and PTB for the 3′ UTR (Shwetha et al., 2015). However, the binding of PTB to the MHV 3′ SL does not affect viral translation (Choi et al., 2002). Similar to the PTB, the cellular factors PABP and PCBP also bind to the 3′ UTR of HCV, NV, and DENV, and regulate viral replication and translation (Herold and Andino, 2001; Tingting et al., 2006; Polacek et al., 2009b; Bailey et al., 2010; Ogram et al., 2010), but the mechanism remains to be studied.

Eukaryotic elongation factor 1A (eEF1A) binds to the stem region close to CS1 in the 3′ UTRs of WNV and DENV (Blackwell and Brinton, 1997; De Nova-Ocampo et al., 2002). The binding of eEF1A to the 3′ UTR is required for WNV and BVDV replication, as confirmed by the co-localization of eEF1A with the WNV and BVDV replication complexes (Johnson et al., 2001; Davis et al., 2007). Notably, eEF1A facilitates WNV replication by enhancing the interaction between the 3′ UTR and the replication complex of WNV (Davis et al., 2007). Nevertheless, eEF1A binds to HCV NS4A and decreases the viral translation efficiency (Kou et al., 2006), suggesting that eEF1A has multiple functions during the replication of members of the *Flaviviridae* family. On the other hand, eEF1A binds to and activate cellular sphingosine kinase 1 (SphK1) (Leclercq et al., 2008), which is involved in inflammatory response and immunomodulation [reviewed in Carr et al. (2013b), Bezgovsek et al. (2018)] and its activity is affected by the DENV-2 3′ UTR (Carr et al., 2013a). Thus, the DENV-2 3′ UTR is postulated to compete with SphK1 for binding to eEF1A and inhibits SphK1 activation, which may be relevant to DENV pathogenicity.

The cellular factors T-cell intracellular antigen-1 (TIA-1) and T-cell intracellular antigen-related protein (TIAR) are important components of stress particles (SGs), which respond to cellular stress responses such as viral infections. TIAR/TIA-1 can be utilized by viruses and localized in viral replication complexes. TIA-1 and TIAR bind to two short sequences consisting of AU in the stem-loop of the negative strand of WNV 3′ UTR and are involved in WNV replication (Li et al., 2002; Mazan-Mamczarz et al., 2006; Emara and Brinton, 2007). Furthermore, TIA-1/TIAR together with the WNV 3′ UTR facilitate the subsequent asymmetric amplification of the viral RNA genome from the minus-strand template, but have little effect on the viral translation efficiency (Emara et al., 2008). Meanwhile, TIA-1/TIAR bind to the TBEV RNA and inhibit viral replication and early viral translation, and this regulation likely determines the amount of RNA available for viral replication and/or assembly (Albornoz et al., 2014). Because both WNV and TBEV are arboviruses, the discrepancy in these results may be attributed to the regulation of viral replication cycle by TIAR/TIA-1. TIAR/TIA-1 is localized in the viral replication complex and may regulates the conversion of virus from low-level symmetric plus- and minus-strand RNA synthesis to asymmetric amplification of plus-strand viral RNA synthesis, thus the role of TIAR/TIA-1 in these two stages may be different (Cleaves et al., 1981; Chambers et al., 1990; Emara et al., 2008). Further experiments are needed to explore the effect of TIAR/TIA-1 on viral replication and translation. In addition, similar findings have also been reported for picornaviruses. For instance, the 3′ UTR of human enterovirus D68 (EV-D68) also interacts with TIA1 (Cheng et al., 2020).

In addition to cellular factors, virus-encoded proteins also interact with viral 3′ stem-loops. Classical swine fever virus (CSFV) encodes the protease NS5A and polymerase NS5B that both bind to the 3′ UTR, which contains two stem-loops, SL-1 and SL-2, and NS5A exhibits higher affinity for binding to the 3′ UTR than the NS5B protein. NS5A binds to SL-1 and SL-2, and the binding of NS5A to SL-1 is more effective than the binding to SL-2; however, NS5B only binds to SL-1 (Sheng et al., 2012). NS5A facilitates viral RNA synthesis at a low concentration, but RNA synthesis is inhibited by a higher concentration of NS5A (Sheng et al., 2010, 2012; Chen et al., 2012). Therefore, it can be hypothesized that a low concentration of NS5A binds to SL-1 along with NS5B and promotes viral RNA replication. However, in the presence of a high concentration of NS5A, the NS5A protein binds SL-2 and SL-1. The binding of NS5A to SL-2 might inhibit viral synthesis by NS5B using the same 3′ UTR as a template. In feline calicivirus (FCV), the nucleolin protein and 3′ UTR bind to and co-localize with viral protease-polymerase NS6 and NS7 proteins in the cytoplasm, and participate in FCV replication (Cancio-Lonches et al., 2011). In addition, the RdRP proteins from encephalomyocarditis virus (EMCV) and duck hepatitis A virus type 1 (DHAV-1) directly bind to the 3′ UTR (Cui et al., 1993; Yu et al., 2017). These results are consistent with the common mechanism used by RdRP from ssRNA(+) viruses to bind to the genomic 3′ UTR and initiate the synthesis of the negative-strand RNA genome (Kok and McMinn, 2009; Lescar and Canard, 2009; Modrow et al., 2013; Paul and Bartenschlager, 2013).

### Pseudoknot Structures

**Figure 6:**
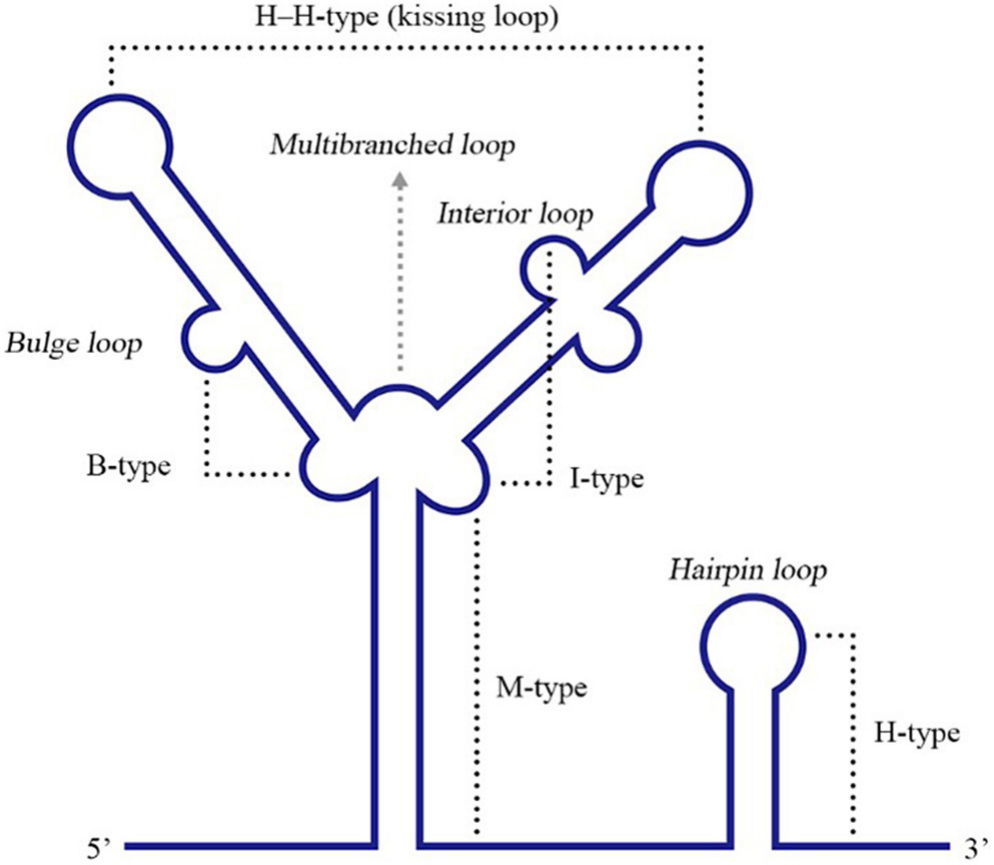
Different types of RNA PK structures. Base pairing between a hairpin loop and a single-stranded region forms a H-type PK, while base paring between a multibranched loop and a single-stranded region forms an M-type PK. A bulge or interior loop together with a multibranched loop can form a B-type or I-type PK. Additionally, two hairpin loops form an H-H-type PK. Adapted from Brierley et al. (2007). This figure has been approved by the original author and obtained the licenses through Rights Licensing Expert (www.copyright.com), the license number was 4045340264208.

Pseudoknots (PKs) are common RNA tertiary structures formed by the complementary pairing of single loops of different stem-loops and are divided into five types, including the hairpin loop and kissing loop (Brierley et al., 2007) (Figure 6). For ssRNA(+) viruses, researches associated with PKs are more common for plant viruses, and PKs used to be called tRNA-1ike structures (TLS) in the early stage since their structural features are similar to tRNAs (Osman and Buck, 2003; Matsuda and Dreher, 2004; Zeenko and Gallie, 2005). Known PKs are widely located in the 5′ UTRs of viral genomes and enhance viral translation (Moes and Wirth, 2007; Easton et al., 2009; Lavender et al., 2010). Nevertheless, PKs in the 3′ UTRs are also indispensable for viral replication and translation.

The DENV 3′ UTR contains two dumbbell structures (DB), which complements with two terminal loops (TLs) sequences of 5 nucleotides, respectively and forms PKs of the hairpin loop type (Olsthoorn and Bol, 2001; Romero et al., 2006) (Figure 3). The base pairing formed by TL2 and PK1, as well as several bases close to TL2, is important for replication and translation (Manzano et al., 2011). The PK sequence is conserved among mosquito-borne flaviviruses. Mutation of PK1 restrains the interaction between the DENV 5′ UTR and 3′ UTR, and mutation of PK2 blocks the complementary interaction of TL2/PK1 (Sztuba-Solinska et al., 2013), suggesting that DENV PK1 and PK2 are functionally interrelated. In addition, the last 285 nucleotides of the 3′ UTR of DENV-2 serve as a “packaging signal,” recruiting NS2A proteins to interact with its PK3, PK4, and 3′ SL to allow the nascent RNA to be recruited from the replication complex to the virion assembly site (Xie et al., 2019).

The HCV NS5B coding region retains several stem-loops containing a 5BSL3.2, which was necessary for HCV replication (Friebe et al., 2005; Diviney et al., 2008; You and Rice, 2008). 5BSL3.2 complements with SL2 in the 3′ UTR and forms a kissing-loop PK, and correct nucleotides pairing in the complementarity region are necessary for efficient HCV replication (Friebe et al., 2005; Diviney et al., 2008). In addition, a 55 nt-long sequence in the X region of the HCV 3′ UTR is completely conserved among all HCV strains, and dimerizes and forms a kissing loop PK in the presence of the virus-encoded core protein (Ivanyi-Nagy et al., 2006). On the other hand, the HCV core protein was reported to bind to the X region (Yu et al., 2009), and mutation analyses showed that dimerization or the PK were relevant to viral replication (Shetty et al., 2010). A logical hypothesis is that the HCV core protein binds to a PK formed by sequences in the 3′ UTR X region and participates in viral replication.

The stem-loop structure of 3′ UTR of some viruses [such as porcine reproductive and respiratory syndrome virus (PRRSV) and equine arteritis virus (EAV)] and viral nucleocapsid protein coding region forms a PK. A highly conserved 34 nt-long sequence of the PRRSV nucleocapsid protein coding region was predicted to fold into a stem-loop and was required for viral RNA replication. Furthermore, 7 nucleotides in the loop region of this stem-loop bind complementarily with a loop in a 3′ UTR stem-loop and form a hairpin loop PK that is necessary for the efficient synthesis of the viral RNA (Verheije et al., 2002). For EAV, the stem-loop structure SL5 of 3′ UTR, together with the stem-loop SL4 of the nucleocapsid protein coding region, form a hairpin loop PK, which is comparatively conserved among arteritis viruses and is relevant to the regulation of viral RNA synthesis (Beerens and Snijder, 2007).

### Other High-Order Structures

The DENV 3′ UTR can fold into two dumbbell structures termed DB1 and DB2 (Figure 3). DENV replication requires the binding of the cellular protein DEAD-box RNA helicase DDX6 to DB1 and DB2 (Ward et al., 2011). Mutations of CS2 and RCS2, which are located in DB1 and DB2, reduce the efficiency of DENV2 translation (Wei et al., 2009). The two dumbbell structures and two terminal loops (TLs) form two PKs, which are important for DENV replication and translation (Olsthoorn and Bol, 2001; Romero et al., 2006; Manzano et al., 2011; Sztuba-Solinska et al., 2013). Moreover, the circularization of the flavivirus genome folds the 5′ UTR and 3′ UTR into a panhandle structure (Villordo and Gamarnik, 2009; Lloyd, 2015), which ensures the efficient initiation of the synthesis of the negative-strand RNA genome. Ochsenreiter et al. (2019), inferred the existence of novel conserved elements in insect-specific flaviviruses (ISFVs) 3′ UTR through covariance models, which may be related to functional importance.

## Other Functions of the 3′ UTR

As shown in Tables 1, 2, most research associated with the ssRNA(+) virus 3′ UTR has focused on viral replication and translation, but the role of the 3′ UTR is by no means limited to these processes. Although numerous studies are not directly based on a certain primary or high-order structure, they have highlighted the important roles of 3′ UTRs in life events of the virus and host.

### Interacting With miRNAs

miRNAs, which are transcribed by the host or virus, are a type of small single-stranded RNAs with the length of ~19–25 nt. They post-transcriptionally regulate the expression level of the target mRNA by forming the RNA-induced silencing complex (RISC). The ssRNA(+) virus genome is directly used as an mRNA to translate viral proteins, which creates conditions for the miRNA to regulate its expression. miRNAs bind to the 5′ UTR, 3′ UTR or coding regions of the viral genome, which is very important for the viral life cycle.

The 3′ UTR of the ssRNA(+) viruses contain miRNA binding sites that interact with the host miRNAs or viral miRNAs to modulate a series of activities in its viral cycle. miR-17 expressed in MDBK cells interacts with the 3′ UTR of bovine viral diarrhea virus (BVDV), enhancing its replication, translation, and RNA stability (Scheel et al., 2016). miR-133a expressed in Vero cells targets the 3′ UTR of the DENV and PTB mRNA in cells. In the early stage of DENV infection, the 3′ UTR of the virus inhibits miR-133a and increases the expression of the PTB protein, which is required for viral replication and translation (Castillo et al., 2016). The 3′ UTR of chikungunya virus (CHIKV) binds to miR-2944b-5p and miR-2b of *Ae. aegypti* cells, but miR-2944b-5p significantly increases viral infection (Dubey et al., 2019). On the other hand, miRNAs have also been reported to suppress viral replication. The 3′ UTR of Eastern equine encephalitis virus (EEEV) contains four miRNA binding sites, namely, three canonical and one non-canonical miR-142-3p sites (Trobaugh et al., 2014). miR-142-3p interacts with the 3′ UTR of EEEV, preventing its replication and translation in myeloid cells (Trobaugh et al., 2014). Mutations at these sites enhance EEEV replication, resulting in higher levels of IFNα/β production, which attenuate viral virulence and prolong the survival of mice (Trobaugh et al., 2019). Interestingly, mosquitoes do not express miR-142-3p, but mutations in the miR-142-3p binding site on the EEEV 3′UTR inhibit virus replication in mosquito cells (Trobaugh et al., 2019). It is speculated other miRNAs in mosquito cells may interact with these binding sites.

### Involvement in sfRNA Formation

In eukaryotic cells, mRNA avoids degradation by endo- and exoribonucleases in the cell through a 5′ cap structure and a 3′ poly(A) tail. However, flaviviruses contain a cap structure at the 5′-end genomic RNA, but do not have poly(A)-tail and terminate with a stem loop structure (3′SL). This 3′SL can protect viral RNA from 3′ to 5′ exoribonucleases, so 5′ to 3′ degradation by exoribonuclease Xrn-1 are likely to be the predominant pathways (Ford and Wilusz, 1999; Narayanan and Makino, 2013).

The genomic RNA of both mosquito- and tick-borne flaviviruses can be digested incompletely by the host's Xrn1 that halts at the Xrn1-resistant RNA (xrRNA) structures within the 3′ UTR and produces a short subgenomic flavivirus RNA (sfRNA). xrRNA appear to be ubiquitously present in many flaviviruses and xrRNA halts diverse exoribonucleases, in addition to Xrn1 (MacFadden et al., 2018; Ochsenreiter et al., 2019). sfRNA has multiple functions, participating in viral cytopathicity and pathogenicity, immune and anti-viral responses of the host and dysregulating endogenous mRNA turnover (Pijlman et al., 2008; Jones et al., 2012; Schnettler et al., 2012, 2014; Chang et al., 2013; Manokaran et al., 2015; Pompon et al., 2017). In addition, sfRNAs have recently been shown to interact with various RNA-binding proteins in cells to regulate RNA decay and splicing (Michalski et al., 2019).

**Figure 7:**
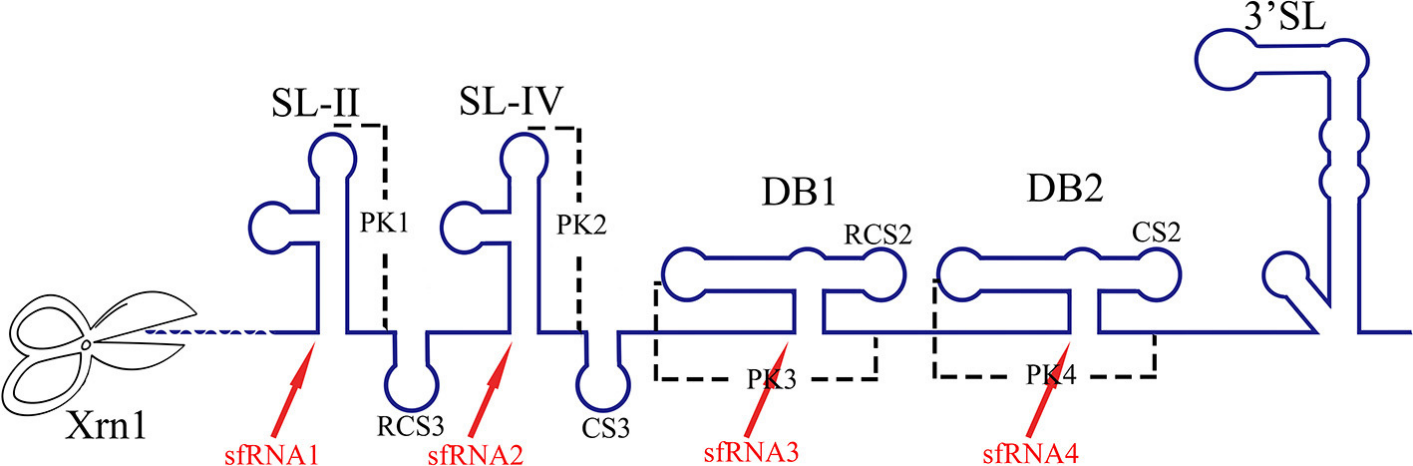
The biogenesis of sfRNA in WNV-infected cells. The primary sequences (RCS3, CS3, RCS2, CS2) and higher order structures (SL-II, SL-IV, DB1, DB2, PK1, PK2, PK3) of the WNV 3′ UTR are required for production of sfRNAs (Pijlman et al., 2008; Funk et al., 2010; Zhang et al., 2020).

The RNA structure at the 3′ UTR is necessary for sfRNA formation (Figure 7). SL-II, SL-IV, DB1, and DB2 in WNV 3′ UTR are required for production of sfRNAs 1–4, respectively (Pijlman et al., 2008; Funk et al., 2010). Recent research shows that RCS3, CS3, RCS2, and CS2 in WNV 3′ UTR are also involved in the production of their corresponding upstream sfRNAs. Among these sequences, RCS3 increases the binding affinity of xrRNA and Xrn1 and stabilizes the three-dimensional structure of the xrRNA (Zhang et al., 2020). Furthermore, the PK is critical for xrRNA function. PK1, PK2, and PK3 blocks degradation of WNV genomic RNA (gRNA), resulting in accumulation of sfRNA (Funk et al., 2010). A similar situation also exists in other flaviviruses. The YFV 3′ UTR was predicted to contain three potential PKs, and researchers have confirmed that pseudoknot 3 (PSK3) serves as the molecular signal to stall Xrn1 and ultimately produce the YFV sfRNA (Silva et al., 2010). Interestingly, Xrn1 knockdown results in a change in the overall ZIKV sfRNA pattern (Akiyama et al., 2016). However, ZIKV sfRNAs inhibit the activity of Xrn1 and disrupt the production of the host mRNA (Michalski et al., 2019). The inhibitory effect of sfRNA on Xrn1 has also been reported in DENV and KUNV (Moon et al., 2012). The production of sfRNA may be a dynamically balanced process, that is to say, once the amount of sfRNA accumulates to a certain level, the activity of Xrn1 will be inhibited and the sfRNAs will exert their regulatory effects.

### Role in Virulence

The effect of the 3′ UTR on virus virulence has been reported. Changes in a certain sequence or structure of the 3′ UTR exert a significant effect on viral virulence. The 3′ UTR poly-pyrimidine sequence affects the virulence of MNV (Bailey et al., 2010). In addition, the 10577T mutation in the WNV 3′ UTR significantly attenuates its virulence (Zhang et al., 2020). The 3′ UTR variable region is the main factor that determines the differential virulence of two strains of TBEV (Sakai et al., 2014), and partial deletion of the secondary structure elements in the 3′ UTR obviously enhanced the virulence, indicating that TBEV virulence is modulated by the secondary structures in the 3′ UTR variable region (Sakai et al., 2015). Similarly, the miR-142-3p binding site on the 3′ UTR of EEEV plays an important role in its virulence (Trobaugh et al., 2019). The impact of the 3′ UTR on virulence has great prospects for the development of live attenuated vaccines.

### Role in Virus Evolution

A small hairpin structure (sHP) (Figure 3) is required for DENV replication in mosquito cells, but not in mammalian cells (Villordo and Gamarnik, 2013); furthermore, DENV RNA structures are relevant to host specialization (Villordo et al., 2015). Similar results have also been reported for Chikungunya virus (CHIKV) and Sindbis virus (SINV), as the 3′ UTRs of these two viruses function as an evolutionary force to adapt to mosquito cells, but not mammalian cells (Chen et al., 2013; Garcia-Moreno et al., 2016). In addition, the sfRNA produced by the flavivirus 3′ UTR participates in regulating host adaptation. DENV generates different patterns of sfRNAs in mosquito or human cells and these patterns rapidly change upon host switching (Filomatori et al., 2017). Similarly, ZIKV infects different cell types to produce different sfRNA patterns (Akiyama et al., 2016). Based on these findings, the sequence and structure of the 3′ UTR must play significant roles in the host adaption and evolution of the ssRNA(+) virus, but the existing data are still very poor and more extensive research is needed.

### Role in the Immune and Anti-viral Responses of the Host

The FMDV 3′ UTR functions as a pathogen-associated molecular pattern (PAMP) and participates in reorganizing and activating the synthesis of the alpha/beta interferon (INF-α/β) mRNA, and the 3′ UTR secondary structure was an important factor contributing to the synthesis of these cytokine (Rodríguez-Pulido et al., 2011). Similarly, the poly-U/UC tract of HCV functions as the PAMP substrate of retinoic acid inducible gene I (RIG-I), since RIG-I was shown to recognize and bind to this region (Saito et al., 2008; Schnell et al., 2012). The infection of DENV2 strain with high epidemiological fitness or the chimeric virus with high epidemiological strain 3′UTR inhibited gene expression for the Toll-pathway component Rel1a and CecG in mosquito salivary glands (Pompon et al., 2017). Moreover, the deletion of the four miR-142-3p binding sites in the 3′ UTR of EEEV result in higher levels of cytokine and chemokine transcription than wild type EEEV (Trobaugh et al., 2019). Thus, specific sequences and structural elements of the ssRNA(+) virus 3′ UTR can mediate the innate immune and anti-viral responses of the host.

In addition, sfRNA produced by 3′UTR has also been reported in antiviral responses. The predominant antiviral innate immune strategy relies on RNA interference (RNAi) pathway in invertebrates (Olson and Blair, 2015). To evade RNAi response, WNV sfRNA efficiently suppressed siRNA- and miRNA-induced RNAi pathways in insect cells (Schnettler et al., 2012). However, when the host becomes a vertebrate, sfRNA can inhibit the production of type 1 interferon. Replication of sfRNA-deficient WNV was rescued in MEFs lacking interferon regulatory factor 3 (IRF-3) and IRF-7 and in mice lacking the type I alpha/beta interferon receptor (IFNAR) (Schuessler et al., 2012). Similarly, IFN antagonist activity of ZIKV, DENV, and JEV sfRNA was also confirmed (Chang et al., 2013; Manokaran et al., 2015; Donald et al., 2016). These results indicate that the study of sfRNA is important for further understanding the pathogenesis and virus-host interaction.

## Perspectives

A certain function is determined by a specific structure, and thus a functional study base on structure is more generally convincing. Here, we describe the primary and higher order structures of the ssRNA(+) virus 3′ UTR, and the function of 3′ UTR for viral replication, translation, virulence, evolution, and the immune response. Therefore, studies of the 3′ UTR are necessary to identify the common mechanisms regulating the life events of all ssRNA(+) viruses.

Life events of the virus and host can be modulated by the interaction between viral 3′ UTR and viral/cellular proteins. On the other hand, the proteins interacting with the 3′ UTR may interact with each other, forming protein-protein interactions, which complement the RNA-protein interactions. These protein-protein and RNA-protein interactions form a large network participating in the regulation of viruses and hosts. However, little is known about these protein-protein interactions, and thus more studies are needed, which will be promising to investigate the complete regulatory network of the ssRNA(+) virus 3′ UTR.

The ssRNA (+) viruses use its genome as mRNA to directly translate viral proteins, which can attract miRNAs transcribed by the host or virus to interact with their genome and regulate the viral life cycle. Given the importance of 3′ UTR to viral replication and translation, the design of an artificial miRNA (amiRNA) targeting the sequence of the 3′ UTR of the ssRNA(+) virus or the insertion of a targeting sequence for the miRNA that inhibits viral replication at the 3′ UTR will provide a new strategy for the treatment of infections with ssRNA(+) viruses. In addition, deleting or mutating certain base sequences in the 3′ UTR disrupts the circularization of the genome, which also provides ideas for the development of new vaccines.

## Author Contributions

YuaL, YZ, and MW conceived, designed, and wrote the manuscript. AC, QY, YWu, RJ, ML, DZ, SC, SZ, XZ, JH, SM, XO, and QG revised with the manuscript. YWa, ZX, ZC, LZhu, QL, YunL, YY, LZha, BT, LP, and XC helped with the manuscript. All authors read and approved the final manuscript for publication.

## Conflict of Interest

The authors declare that the research was conducted in the absence of any commercial or financial relationships that could be construed as a potential conflict of interest.
